# Metabolic Profile Analysis of Designer Benzodiazepine Etizolam in Zebrafish and Human Liver Microsomes

**DOI:** 10.3390/metabo13060699

**Published:** 2023-05-27

**Authors:** Zhaowei Jie, Shiyang Qin, Wenfang Zhang, Jifen Wang, Jianghai Lu, Ge Qin, Xiaolong Hou, Peng Xu

**Affiliations:** 1 School of Investigation, People’s Public Security University of China, Beijing 100038, China; jiezhaowei@outlook.com (Z.J.); qg18263373596@outlook.com (G.Q.); houxiaolong930@gmail.com (X.H.); 2 Forensic Science Service of Beijing Public Security Bureau, Key Laboratory of Forensic Toxicology, Ministry of Public Security, Beijing 100192, China; qsy19831029@gmail.com (S.Q.); zwf13911208706@outlook.com (W.Z.); 3 Drug and Food Anti-Doping Laboratory, China Anti-Doping Agency, 1st Anding Road, Beijing 100029, China; 4 Key Laboratory of Drug Monitoring, Control and Anti Drug Key Technologies, Ministry of Public Security, Anti Drug Information Technology Center of the Ministry of Public Security, Beijing 100193, China; xupeng12345609@outlook.com

**Keywords:** Etizolam, metabolic profile, zebrafish, human liver microsomes, metabolic pathways

## Abstract

As one of the most widely abused designer benzodiazepines worldwide, Etizolam is characterized by its high addiction potential, low production cost, and difficulty in detection. Due to the rapid metabolism of Etizolam in the human body, the probability of detecting the Etizolam parent drug in actual case samples by forensic personnel is low. Therefore, without detecting the parent drug, analysis of Etizolam metabolites can help forensic personnel provide references and suggestions on whether the suspect has taken Etizolam. This study simulates the objective metabolic process of the human body. It establishes a zebrafish in vivo metabolism model and a human liver microsome in vitro metabolism model to analyze the metabolic characteristics of Etizolam. A total of 28 metabolites were detected in the experiment, including 13 produced in zebrafish, 28 produced in zebrafish urine and feces, and 17 produced in human liver microsomes. The UPLC-Q-Exactive-MS technology was used to analyze the structures and related metabolic pathways of Etizolam metabolites in zebrafish and human liver microsomes, and a total of 9 metabolic pathways were identified, including monohydroxylation, dihydroxylation, hydration, desaturation, methylation, oxidative deamination to alcohol, oxidation, reduction acetylation, and glucuronidation. Among them, metabolites involving hydroxylation reactions (including monohydroxylation and dihydroxylation) accounted for 57.1% of the total number of potential metabolites, indicating that hydroxylation may be the major metabolic pathway of Etizolam. Based on the response values of each metabolite, monohydroxylation (M1), desaturation (M19), and hydration (M16) were recommended as potential biomarkers for Etizolam metabolism. The experimental results provide reference and guidance for forensic personnel in identifying Etizolam use in suspects.

## 1. Introduction

In recent years, with the development of the international drug situation, new psychoactive substances have quietly emerged as the third generation of drugs. Designer benzodiazepines are defined as new psychoactive substances containing the fused ring structure of benzodiazepine, and these drugs are gradually becoming the newest severe psychoactive substances abused worldwide due to their highly addictive, low-cost, and difficult-to-detect characteristics [1]. Etizolam (4-(2-chlorophenyl)-2-ethyl-9-methyl-6H-thieno[3,2-f][1,2,4]triazolo [4,3-a][1,4]diazepine) is a thieno-benzodiazepine derivative, which differs from traditional designer benzodiazepines in that a thieno ring replaces the benzene ring in the parent nucleus. Etizolam, the most popular designer benzodiazepine today, has been detected in many cases worldwide [2,3,4,5,6]. According to the report of the United Nations Office on Drugs and Crime in 2022, benzodiazepine-type substances appeared in 47% of post-mortem drug testing cases and 67% of drug-driving cases involving dangerous drugs, and cases involving Etizolam accounted for about 35% of the total number of these two types of cases, which is higher than the other designer benzodiazepines.

Currently, some progress has been made in detecting Etizolam and its metabolites. There have also been reports on the metabolic patterns of Etizolam in vivo and in vitro. Balkhi [7] et al. simulated the in vitro metabolism of Etizolam using human liver microsomes and identified five metabolites. Erin [8] et al. studied the metabolites of Etizolam in horses using two metabolic models, namely, horses and horse liver microsomes, and identified 23 metabolites with α-OH-Etizolam as a potential metabolic marker. Despite the numerous reports on the metabolic studies of Etizolam in vivo and in vitro, the metabolic profile of Etizolam is still not comprehensive, and the metabolic pathways are limited to hydroxylation, hydration, and desaturation [9,10,11]. Moreover, there is a lack of characteristic fragment analysis of Etizolam and its metabolites in the mass spectrometry field. Therefore, it is essential and urgent to analyze the metabolic profile of Etizolam in vivo and in vitro and conduct corresponding characteristic fragment mass spectrometry analysis.

Zebrafish and human liver mitochondria are two mature metabolic models widely used in preliminary studies of new psychoactive substance metabolism [12,13]. As a model animal, zebrafish has advantages over other metabolic models, such as small size, easy maintenance, and low cost. Moreover, zebrafish have a high similarity of up to 70% with human genes [14,15,16]. They can present metabolic processes similar to mammals, which supports the use of zebrafish models in NPS metabolism research. The liver mitochondria model, the most widely used in vitro metabolic model in drug metabolism, has the characteristics of simple preparation, solid metabolic ability, and good reproducibility. However, the application of zebrafish models in designer benzodiazepines-related metabolism research is minimal [17,18,19]. Currently, only Kong [20] et al. have used zebrafish metabolic models to simulate the in vivo metabolism process of Clonazolam, and a total of seven I-phase metabolites and one II-phase metabolite were found in the experiment, which provides support for the feasibility study of zebrafish models in designer benzodiazepines metabolism to some extent.

Our study aims to analyze the metabolic profile of Etizolam using two metabolic models, zebrafish and human liver microsomes. Liquid chromatography-high resolution mass spectrometry (LC-HRMS) will be employed to identify the metabolites of Etizolam and its related metabolic pathways in vivo and in vitro. Potential metabolic biomarkers will be determined based on signal intensity and detection duration, and the time pattern of related metabolites will be explored. Ultimately, this study will provide valuable references for the forensic identification of whether suspects have taken Etizolam.

## 2. Experimental Section

### 2.1. Instruments and Reagents

The Vanquish Duo ultra-high-performance liquid chromatography system coupled with a Q-Exactive hybrid quadrupole-orbitrap mass spectrometer (Thermo Fisher Scientific, Bremen, Germany); Mettler Toledo AL104 electronic balance (Mettler Toledo, Zurich, Switzerland); Bio Tool D34135R low-temperature centrifuge (Bio Tool, Kirchberg, Switzerland); IKA VORTEX2 vortex mixer (IKA, Bremen, Germany); and ball mill (Zhejiang Di’anjian Scientific Instrument Co., Ltd., Zhejiang, China) were used in this study. The Etizolam standard (1 mg/mL) was provided by the Key Laboratory of Forensic Toxicology Analysis of the Ministry of Public Security. Methanol (chromatography grade, Merck, Darmstadt, Germany), formic acid (HPLC grade, Dikmapure, Beijing, China), acetonitrile (chromatography grade, Merck, Darmstadt, Germany), solid phase extraction column (Waters Oasis WCX 3cc, Waters, New York, NY, USA), and ultrapure water (Watsons Water, China) were used. Human liver microsomes (HLMs, provided by Beijing Huizhi Taikang Co., Ltd., Beijing, China) with a protein concentration of 20 mg/mL, NADPH regeneration system (NADPH type-A and NADPH type-B, Provided by Beijing Huizhi Taikang Co., Ltd., Beijing, China), 0.1 mol/L phosphate buffer (provided by Beijing Huizhi Taikang Co., Ltd., Beijing, China), and uridine diphosphate glucuronic acid trisodium salt (UDPGA, provided by Beijing Huizhi Taikang Co., Ltd., Beijing, China) were also used.

### 2.2. Chromatographic Condition

Mobile phase A was a 0.1% aqueous solution of formic acid containing 10 mmol/mL ammonium formate, while mobile phase B was a methanol solution containing 0.1% formic acid. The injection volume was 5 μL, and the flow rate was 0.5 mL/min. A Thermo Hypersil GOLD column (100 mm × 2.0 mm, 1.7 μm) was used at a temperature of 30 °C. A gradient elution method was employed as follows: 5% B for 0–0.5 min, 5–30% B for 0.5–6.5 min, 50% B for 6.5–9.5 min, 30% B for 9.5–13.5 min, 50% B for 13.5–22 min, 50–95% B for 22–22.01 min, 95% B for 22.01–26.50 min, 95–5% B for 26.50–26.51 min, and 5% B for 26.51–28.00 min.

### 2.3. Mass Spectrometry Conditions

Ion source: electrospray ionization source (positive ion mode) with a spray voltage of 4 kV; ion source temperature: 330 °C, ion transfer tube temperature: 370 °C; resolution: 60,000; automatic gain control (AGC): 1 × 105; and scan range set to 100–1000 *m/z*.

### 2.4. Establishment of Zebrafish In Vivo Metabolism Model

Adult healthy zebrafish (3–6 months old; 0.17–0.6 g, Beijing Huante Biological Zebrafish Breeding Laboratory) of both sexes were selected within a quality range of 1 ± 0.5 g and divided into two experimental groups (3 fish per group) and one blank control group (6 zebrafish without Etizolam). The experimental groups were exposed to ultrapure water containing 5 ug/mL Etizolam. After 4 h and 54 h, 3 zebrafish were removed from each group, and the corresponding fish tank water was extracted. The blank control group was placed in ultrapure water, and after 4 h and 54 h, 3 zebrafish were sequentially taken out from each group, and the corresponding fish tank water was extracted. After the culture process, zebrafish were rinsed with water, placed in grinding tubes, and euthanized with 300 µL acetonitrile. The samples were ground using a ball mill with the following parameters: frequency: 60 Hz; cycle times: 6; running time: 45 s; and interruption time: 15 s. After grinding, we took out the sample and divided it into two equal parts. One part was placed in a solid-phase extraction column for solid-phase extraction, and the extraction liquid was subjected to nitrogen blowing and redissolution. Finally, the samples were taken out and centrifuged at 5 °C for 10 min, and then 100 µL of the supernatant was filtered through a 0.22 μm PTFE microporous membrane for injection analysis. The other portion was centrifuged directly at 5 °C for 10 min, and then 100 µL of the supernatant was filtered through a 0.22 μm PTFE microporous membrane for injection analysis. All experimental animal procedures followed the regulations of the Key Laboratory of Drug Monitoring and Control and the Key Laboratory of Drug Prohibition Technology of the Ministry of Public Security and were approved by the Animal Ethics Committee for Experimental Animals (Approval No.: KLDMC-WECLA-202212-01).

### 2.5. Establishment of Human Liver Microsome Metabolism Model

Etizolam was dissolved in methanol to prepare a standard solution of 1 mg/mL. An amount of 2 μL of the standard solution was added to the newly prepared incubation system liquid to make the final system volume 200 μL (including 10 μL of NADPH-SolutionA, 2 μL of NADPH-SolutionB, 20 μL of UDPGA (50 mM), 20 μL of Alamethicin (250 ug/mL), 20 μL of D-Saccharic acid 1,4lactonemonohydrate (50 mM), 10 μL of human liver microsomes (20 mg/mL), and 116 μL of 0.1 M buffer solution). Four liver microsome preparations were made under the same conditions. The regeneration system initiated the metabolic reaction and was carried out at 37 °C in a water bath. The reaction time was recorded using a stopwatch, and at 30 min, 45 min, 60 min, and 90 min, one liver microsome was taken out successively. The reaction was terminated by adding 200 μL of ice-cold acetonitrile. After the reaction was terminated, the liver microsomes were centrifuged at 14,000 r/min at 4 °C for 10 min. An amount of 100 μL of the supernatant was transferred to a glass sample bottle for scanning analysis. The blank solution, incubation reaction system solution without the target drug, reaction system solution without liver microsomes, and reaction system solution without the regeneration system were analyzed as control groups.

### 2.6. Identification Strategy

The experiment utilized UPLC-Q-Exactive-MS to scan the metabolites generated by Etizolam in zebrafish and human liver microsomes at different time intervals. The raw signals obtained from the scans were imported into Compound Discover 2.1 for analysis, and the characteristic fragment ion structures of the assumed metabolites were predicted. Based on the appearance patterns of these fragment structures, the metabolites’ metabolic pathways and corresponding structural formulas were deduced. The experiment provided accurate masses for all the results (retained to five decimal places). The identification strategy for the metabolites was as follows: (1) the mass error of protonated molecules was less than (2) the diagnostic product ions, which had appropriate peak shapes, retention times, and MS/MS spectra, and no corresponding spectra were found in the negative control group; (3) the comparison of the characteristic fragment ion *m/z* values of each metabolite with Etizolam enabled the determination of the position of each metabolic site and the hypothetical structure of each metabolite; (4) potential metabolic biomarkers were determined based on the chromatographic peak area, signal intensity, and detection duration. Based on the determined structures of the metabolites, the metabolic pathways associated with Etizolam were proposed.

## 3. Results and Discussion

### 3.1. Selection and Optimization of Experimental Methods

#### 3.1.1. Selection and Optimization of NCE Value for Instrumentation

This study utilized the UPLC-Q-Exactive-MS instrument to perform complete MS/dd-MS^2^ analysis on zebrafish and liver mitochondria samples, generating corresponding first- and second-level mass spectrometry fragments. The selection of the mass spectrometer’s second-level collision energy (NCE) directly affects the information richness of the second-level mass spectrum. If the NCE value is higher, the degree of dissociation of ions during collision is high, resulting in fewer ion fragments in the second-level mass spectrum, which is not conducive to the structural analysis of relevant substances. Conversely, the NCE value is too high. In that case, the precursor ion of the metabolite will undergo complete dissociation after being accelerated to a specific energy, and no corresponding precursor ion peaks will appear in the second-level mass spectrum. Therefore, selecting an appropriate NCE value is essential for the second-level mass spectrometry analysis of the parent drug and related metabolites.

**Figure 1:**
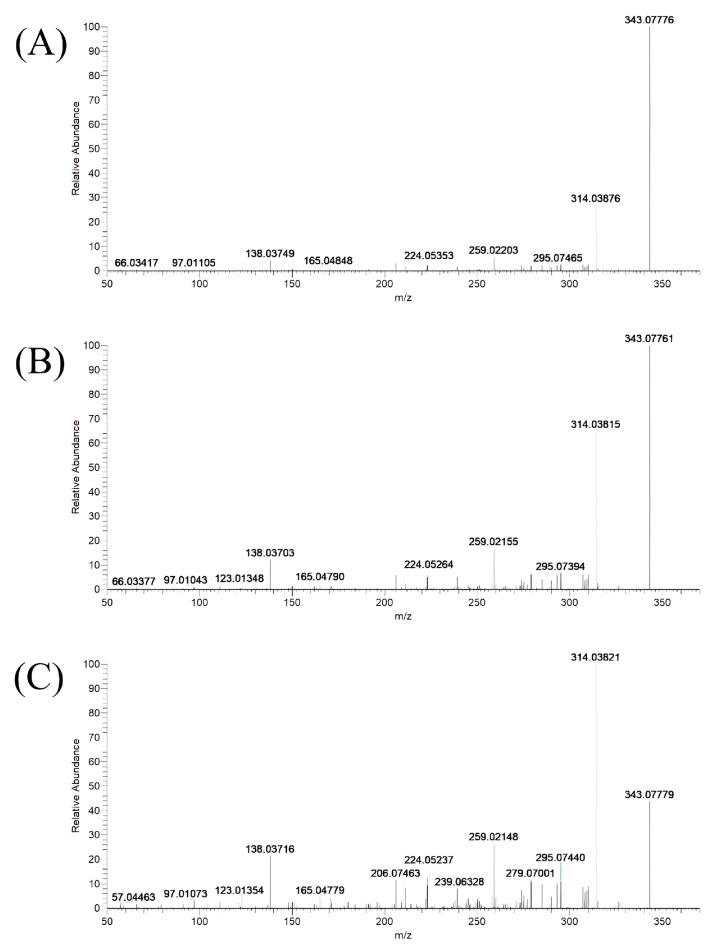
MS/MS spectra of Etizolam under different collision energies. (**A**) Average NCE value: 40; (**B**) average NCE value: 50; and (**C**) average NCE value: 60.

The experimental procedure explored three different Stepped NCE values: (1) 10, 40, 70 (with an average NCE value of 40), (2) 30, 50, 70 (with an average NCE value of 50), and (3) 40, 60, 80 (with an average NCE value of 60). The effect of these three NCE values on the MS/MS spectra was compared (Figure 1), with the criterion for evaluating the level of MS/MS spectra refinement being the appearance of as many fragment ions as possible while maintaining the appearance of the parent ion peak. Figure 1 shows that the Stepped NCE values of 40, 60, and 80 produced the best fragmentation efficiency while ensuring the appearance of the parent ion peak. Therefore, the experiment was conducted under the conditions of Stepped NCE: 40, 60, 80 (with an average NCE value of 60) to analyze the MS/MS fragments of Etizolam and its related metabolites, which improved the accuracy and sensitivity of the analysis results.

#### 3.1.2. Selection of Pre-Treatment Methods for Zebrafish Experiment

Solid phase extraction (SPE) [21,22,23,24] and protein precipitation technology (PPT) are widely used technologies in the field of toxicology. The advantages of SPE are its ability to simultaneously enrich and purify the sample, effectively improving the sensitivity of instrument detection. Additionally, this method has good reproducibility and provides stable sample results when processed with this method. Protein precipitation technology is a commonly used method for sample pre-treatment in toxicological analysis. This technique removes interfering proteins from biological samples that may affect the accuracy of the analysis while keeping the targeted toxic substance in solution. The significance of this method lies in its ability to improve the analysis environment and enhance the reliability of instrument detection results.

The experiment employed solid-phase extraction and protein precipitation techniques to select and compare the pre-processing methods for zebrafish experiments. Combined with the identification strategy, the study summarized the types of metabolites found in zebrafish and urine after applying the two pre-processing methods (Table 1). Table 1 demonstrated that 29 metabolites were identified in zebrafish and urine after pre-processing with solid-phase extraction, while only 14 were identified after pre-processing with protein precipitation. The experimental results indicated that selecting solid-phase extraction as the pre-processing method enabled the identification of more metabolites in zebrafish, which was more conducive to exploring the metabolic regulation of Etizolam in zebrafish and obtaining a more evident metabolic profile.

### 3.2. Etizolam Fragmentation

**Figure 2:**
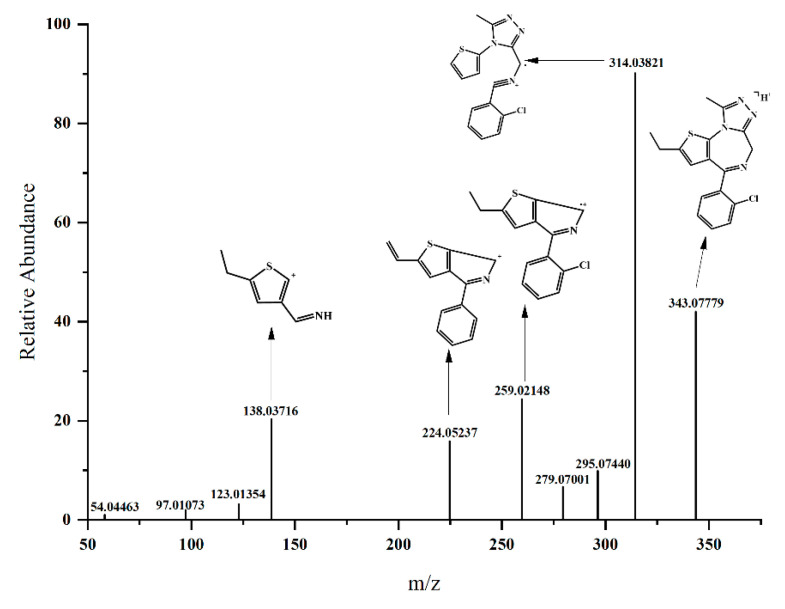
Analysis of fragment characteristics in Etizolam’s secondary mass spectrometry.

The analysis of characteristic fragments of the original drug is of great significance for the structural analysis of related metabolites. Etizolam was eluted at 19.70 min (*m*/*z* 343.07779, C17H15N4ClS) (Figure 2) and generated four characteristic fragment ions after fragmentation: (1) *m*/*z* 314.03821 (C15H11N4ClS) (elimination of the ethyl group on the thiophene ring and rearrangement of the diazepine ring); (2) *m*/*z* 259.02148 (C14H11NClS) (elimination of the imidazole ring and part of the diazepine ring); (3) *m*/*z* 224.05237 (C14H10NS) (elimination of the imidazole ring and part of the diazepine ring, and the ethyl group becomes a vinyl group); and (4) *m*/*z* 138.03716 (C7H8NS) (elimination of the chlorobenzene ring, imidazole ring, and part of the diazepine ring).

### 3.3. Identification of Etizolam metabolites

**Figure 3:**
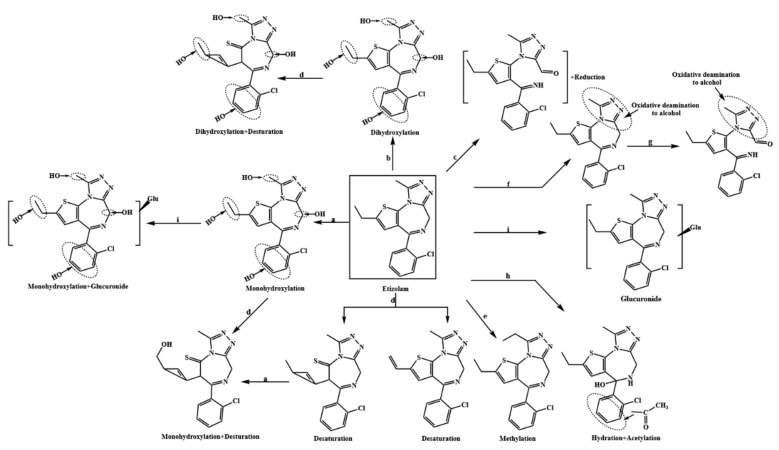
Postulated metabolic pathway of Etizolam. a: monohydroxylation; b: dihydroxylation; c: hydration; d: desaturation; e: methylation; f: oxidative deamination to alcohol; g: oxidation; h: reduction + acetylation; and i: glucuronide.

A total of 29 Etizolam metabolites were detected in both zebrafish in vivo metabolic model and human liver microsomes in vitro metabolic model, involving 9 metabolic pathways, including 25 phase I metabolites and 4 phase II metabolites. The mass deviations of all detected metabolites were within 5 ppm, and Etizolam parent drug or degradation products were not detected in the blank control group. Etizolam was labeled as P, and its metabolites were labeled as M. The retention time, mass deviation, elemental composition, and characteristic ion fragments information of all detected metabolites were summarized (Table 2), and the metabolic profile of Etizolam was obtained (Figure 3).

According to Table 2, 13 Etizolam metabolites were found in zebrafish homogenate, 28 metabolites were found in zebrafish urine and feces, and a total of 17 metabolites were found in human liver microsomes. Among them, there were 15 metabolites that have not been reported in the literature. The newly discovered metabolic pathways were mainly concentrated in oxidative degradation to alcohol, reduction + acetylation, and oxidation.

#### 3.3.1. Monohydroxylation

**Figure 4:**
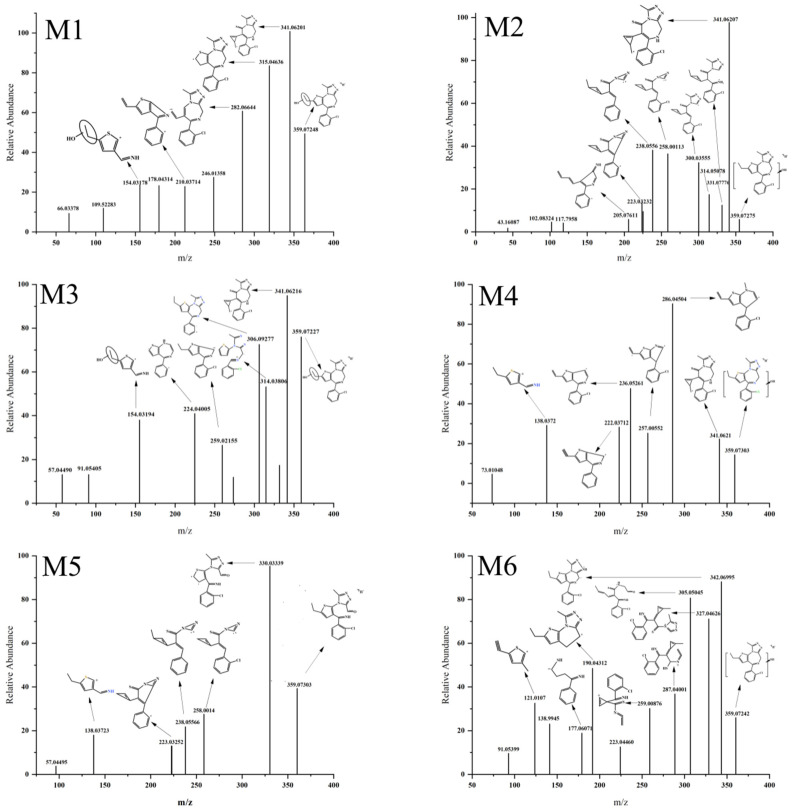

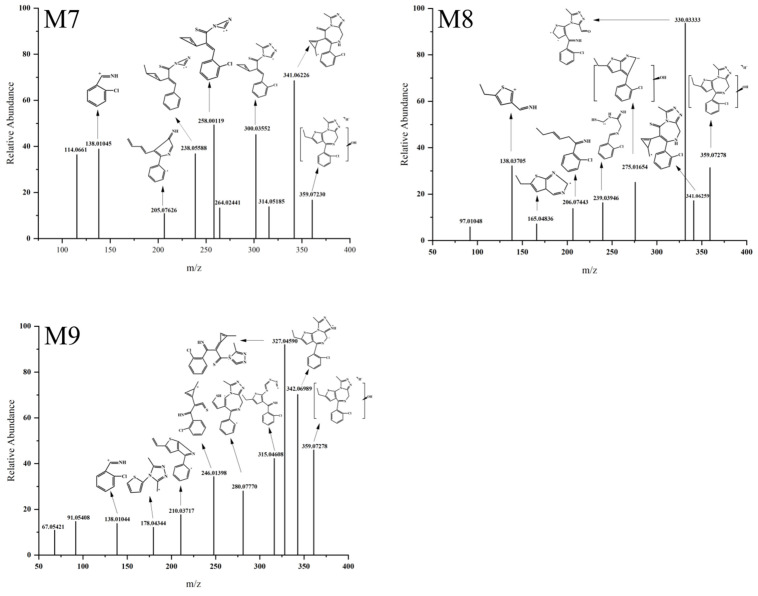
MS/MS spectra of Etizolam monohydroxylated metabolites.

The [M H]^+^ values of M1, M2, M3, M4, M5, M6, M7, M8, and M9 were 359.07279 (Figure 4), which is 15.99526 Da larger than the parent drug [M H]^+^, indicating that a hydroxylation reaction occurred. M1 and M3 generated hydroxylation fragment ions at *m/z* 154.031 (Figure 4). Since the enol structure formed by the double-bonded carbon atom in the thiophene ring and the hydroxyl group cannot exist stably, it is believed that the binding site of the hydroxylation reaction occurred in the ethyl part connected to the thiophene ring.

M5 and M8 generated fragment ions at *m/z* 330.033 (Figure 4), indicating that after the hydroxylation reaction occurred at the carbon atom site on the diazepine ring, two situations occurred. One is that the hydroxyl group replaced the hydrogen atom on the internal carbon atom of the diazepine ring, forming a stable hydroxylated metabolite. The other situation is that the introduction of the hydroxyl group caused hydrolysis of the diazepine ring, followed by oxidation of the alcohol hydroxyl group to an aldehyde group. It is worth noting that M8 generated fragment ions at *m/z* 341.06259 (Figure 4), indicating that no ring-opening hydrolysis reaction occurred in the diazepine ring of M8, corresponding to the first situation mentioned above, while M5 did not have corresponding fragment ions, corresponding to the second situation mentioned above.

**Figure 5:**
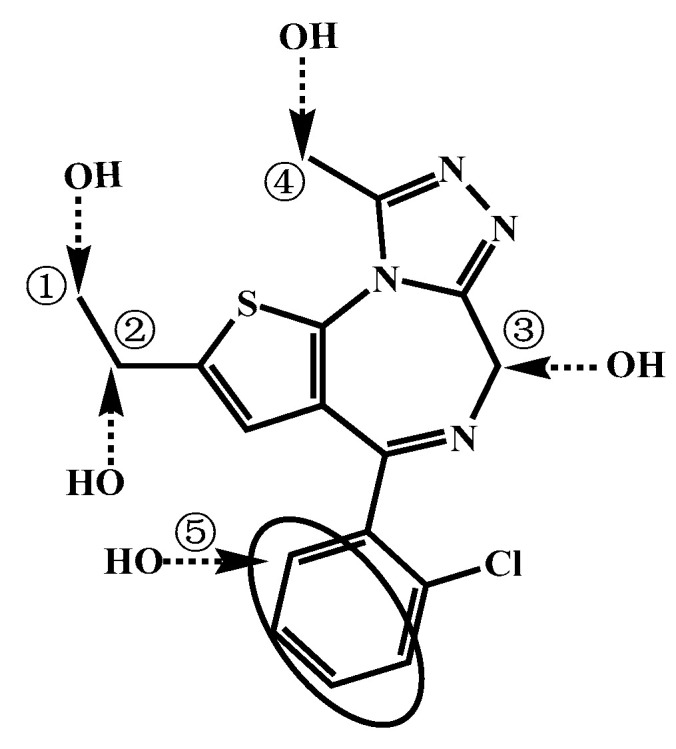
Possible hydroxylation sites in Etizolam.

It is speculated that the hydroxylation sites of M2, M4, M6, M7, and M9 occurred in the benzene ring and the methyl part connected to the imidazole ring. The reason for this analysis is that these five metabolites did not generate ion fragments at *m/z* 330.033 and *m/z* 154.031, ruling out the possibility of hydroxyl groups being located on the ethyl or diazepine ring. According to the Etizolam structure (Figure 5), it is most likely that the hydroxylation sites in M2, M4, M6, M7, and M9 occurred in the benzene ring (⑤ in Figure 5) or the methyl part connected to the imidazole ring (④ in Figure 5).

#### 3.3.2. Dihydroxylation

**Figure 6:**
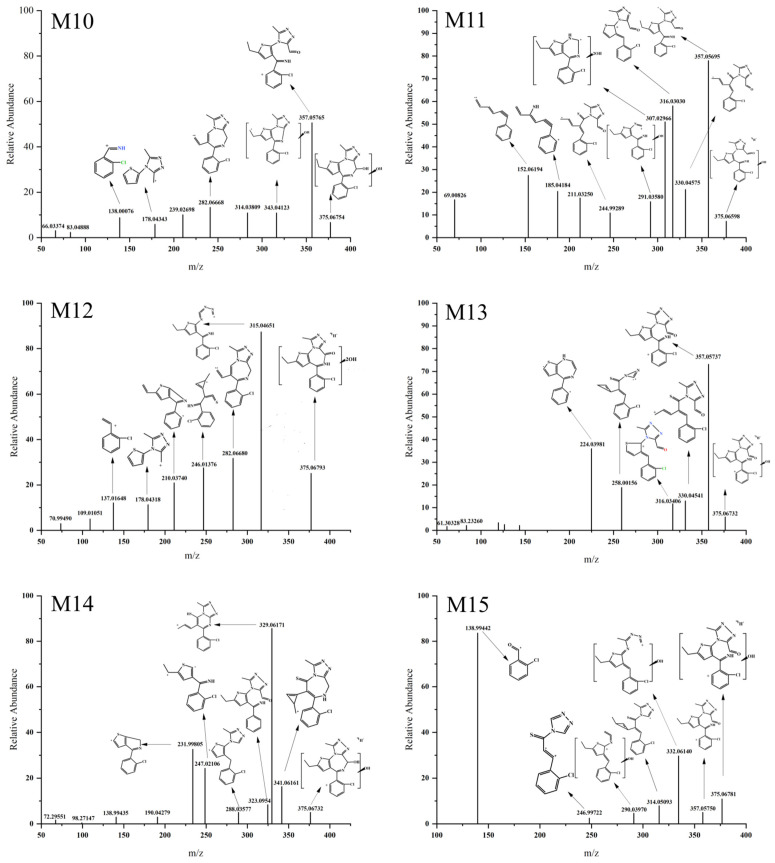
MS/MS spectra of Etizolam dihydroxylation metabolites.

The [M H]^+^ values of M10, M11, M12, M13, M14, and M15 were 375.06770 (Figure 6), indicating that dihydroxylation reaction had occurred with an increase of 31.99017 Da compared to the parent drug [M H]^+^. Fragment ions were generated at *m/z* 357.05 (Figure 6) in M10, M11, M13, and M15, suggesting that one hydroxyl group was attached to a carbon atom ③ (Figure 5) of Etizolam. In contrast, the other hydroxyl group was attached to positions ①, ②, ④, and ⑤ (Figure 5). M11 generated a hydroxylation fragment ion at *m/z* 307.02996, eliminating the possibility of the other hydroxyl group attaching to a carbon atom ④. M12 and M14 generated ion fragments at *m/z* 282.06680 and *m/z* 341.06161, respectively, indicating that the imidazole ring did not undergo a ring-opening rearrangement, and the possibility of hydroxyl groups attaching to the imidazole ring was low. Therefore, the two hydroxyl groups were most likely attached to positions ①, ②, ④, and ⑤ (Figure 5).

#### 3.3.3. Hydration and Desaturation

**Figure 7:**
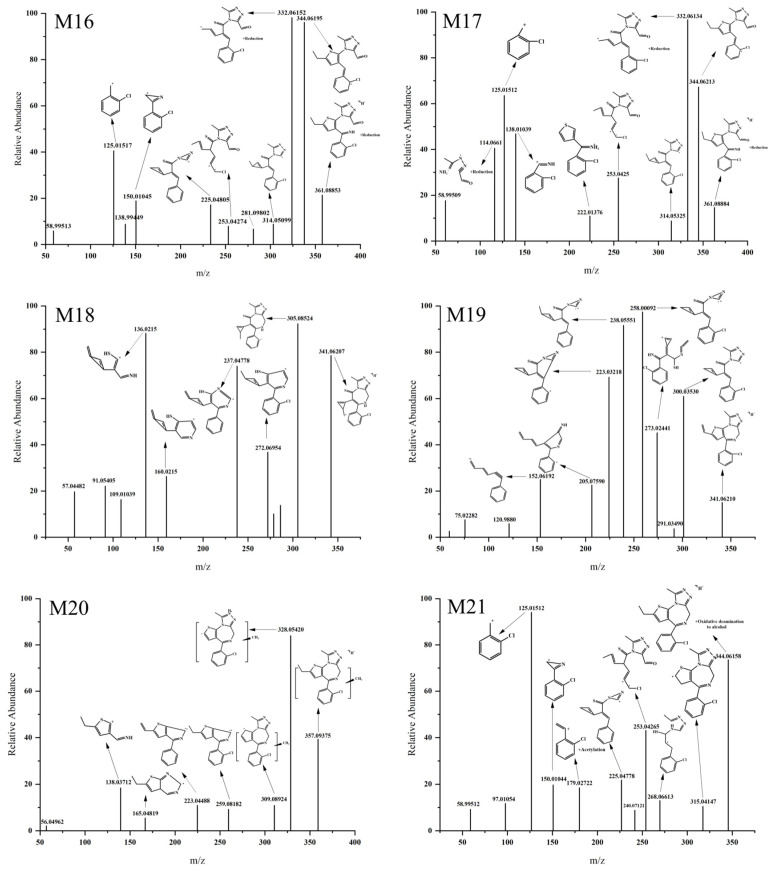
MS/MS spectra of Etizolam hydrated and desaturated metabolites.

The [M H]^+^ values of M16 and M17 were 361.088 (Figure 7), which is 18.01047 Da larger than that of the parent drug, indicating the occurrence of a hydration reaction. The ion fragments were produced at *m/z* 344.06, *m/z* 332.061, and *m/z* 314.05 in M16 and M17. Based on the MS/MS spectra of M16 and M17 (Figure 7), it can be inferred that the hydroxyl group in the hydration reaction was bonded to the carbon atom in the diazaphenanthrene ring, followed by a ring-opening and rearrangement to form an aldehyde group. It is worth noting that the difference between M16 and M17 lies in the different positions of hydrogen atom reduction. M16 did not produce fragment ions at *m/z* 253.0425. However, it produced a fragment ion peak at *m/z* 332.06152, indicating that the hydrogen atom reduction most likely occurred at the carbon–oxygen double bond of the aldehyde group. M17 produced a fragment ion peak at *m/z* 114.0661, suggesting that the reduction position of the hydrogen atom was at the double or triple bond in the fragment ion at *m/z* 114.0661.

The [M H]^+^ values of M18 and M19 were 341.062 (Figure 7), which is 2.01552 Da smaller than that of the parent drug, indicating the occurrence of a desaturation reaction. M18 produced fragment ions at *m/z* 305.08524, *m/z* 272.06954, *m/z* 237.04778, and *m/z* 136.0215. Based on the MS/MS spectrum of M18 (Figure 7), it can be inferred that the thieno ring underwent ring-opening and rearrangement to form a new three-membered ring. Through the fragment ion structures at *m/z* 205.07590 and *m/z* 152.06192, it is speculated that the ethyl group connected to the thieno ring in M19 underwent a desaturation reaction, forming a carbon–carbon double bond.

#### 3.3.4. Methylation and Oxidative Deamination to Form Alcohols

The [M H]^+^ value of M20 was 357.09375 (Figure 7), which is 14.01622 Da larger than that of the parent drug, suggesting the occurrence of a methylation reaction. The possibility of methylation combined with the ethyl group on the thieno ring was excluded by the two ion fragments at *m/z* 328.05420 and *m/z* 309.08924, and the possibility of methylation connected to the phenyl ring was ruled out by the presence of the ion fragment at *m/z* 259.08182. Therefore, it is speculated that the methylation site is located on the methyl group connected to the imidazole ring.

The [M H]^+^ value of M21 was 344.06158 (Figure 7), which is 0.98405 Da larger than that of the parent drug. Combined with Compound Discover 2.1, it is speculated that M21 underwent oxidative deamination to form an alcohol. The fragment ion structure at *m/z* 179.02722 indicates that the deamination occurred at the imidazole ring.

#### 3.3.5. Hydration and Acetylation

**Figure 8:**
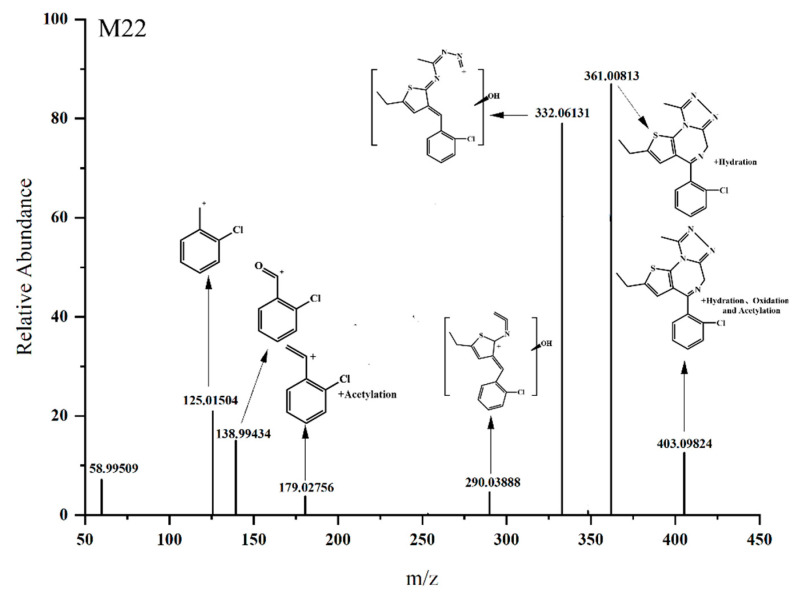
The MS/MS spectra of the hydration and acetylation metabolites of Etizolam.

The [M H]^+^ value of M22 was 403.09824 (Figure 8), which is 60.02071 Da larger than that of the parent drug, suggesting the occurrence of hydration and acetylation reactions. Based on the ion fragment at *m/z* 179.02756, it is speculated that the acetylation site is located on the phenyl ring. Combined with the ion fragments at *m/z* 361.00813 and *m/z* 344.06152, it is inferred that the hydration reaction occurred inside the diazaphenanthrene ring, and according to the structure at *m/z* 344.06152, the hydration reaction most likely occurred at the carbon–nitrogen double bond in the diazaphenanthrene ring.

### 3.4. Exploration of the Metabolism Pattern of Etizolam

**Figure 9:**
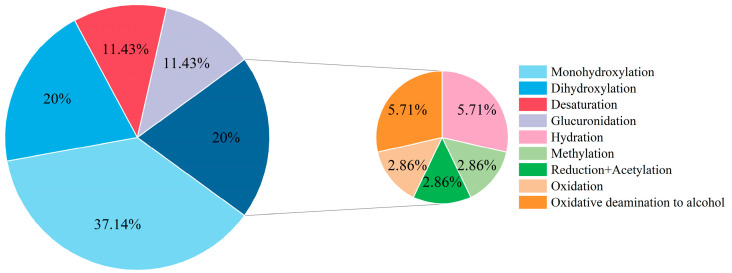
Proportions of 29 Etizolam metabolites in metabolic pathways.

Based on the experimental results, 29 metabolites were identified in Etizolam’s in vivo and in vitro metabolism models in zebrafish and human liver microsomes, involving 9 metabolic pathways. The metabolic pathways involved in the 29 metabolites in Table 2 were analyzed and statistically summarized (Figure 9). The results showed that metabolites involving hydroxylation reactions (including monohydroxylation and dihydroxylation) had the highest proportion, accounting for approximately 57.1% of the total potential metabolites. The reasons for this are twofold: (1) the structure of Etizolam provides multiple hydroxylation binding sites (Figure 5), and hydroxylation can generate stable hydroxylated metabolites after binding to these sites; (2) the presence of the Cytochrome P450 enzyme system (CYP450) makes hydroxylation reactions easier to occur. CYP450 is a heme-containing monooxygenase that activates molecular oxygen, allowing one oxygen atom to bind to an organic molecule while reducing the other to water. When Etizolam enters zebrafish and human liver microsomes, the catalytic action of CYP450 accelerates the formation of OH-Etizolam. Notably, compared with the metabolism studies of other designer benzodiazepines [25,26,27,28,29], three new metabolic pathways were identified: oxidative deamination to alcohol, reduction acetylation, and desaturation. The analysis suggests that these three metabolic pathways are strongly related to Etizolam’s structure, which is a derivative of thienodiazepine and has a thieno ring that replaces the benzene ring in the traditional nucleus of benzodiazepines, resulting in significant changes in its pharmacological properties. Based on the response values of each metabolite, monohydroxylation (M1), desaturation (M19), and hydration (M16) were recommended as potential biomarkers for Etizolam metabolism.

## 4. Conclusions

This study used zebrafish and human liver microsome models to investigate Etizolam in vivo and in vitro metabolism. The UPLC-Q-Exactive-MS technology was utilized to analyze the structures of the metabolites produced by Etizolam, the involved metabolic pathways, and the potential toxicological features of the drug. The experimental results showed that 28 metabolites were produced in the zebrafish system and 14 were produced in human liver microsomes. Among the 28 metabolites identified, monohydroxylation (M1), desaturation (M19), and hydration (M16) were recommended as potential biomarkers for Etizolam use, as these characteristic chemical structures retained more of the parent nucleus and had high response intensities in both zebrafish and human liver microsome metabolism models. In addition, among the potential metabolites identified, metabolites involving hydroxylation reactions had the highest proportion, indicating that hydroxylation may be the major metabolic pathway of Etizolam.

The current study highlights the practicality and effectiveness of zebrafish and human liver microsome metabolism models in analyzing the metabolism of NPS. The present study provides reference and evidence for forensic personnel in identifying the use of Etizolam, but more cases are needed to validate our findings.

## Figures and Tables

**Table 1:** Summary of the types of metabolites detected in zebrafish tissues and urine using two different pre-processing methods.

ID	Biotransformation	SPE	PPT
M1	Monohydroxylation	Y	Y
M2	Monohydroxylation	Y	N
M3	Monohydroxylation	Y	N
M4	Monohydroxylation	Y	Y
M5	Monohydroxylation	Y	Y
M6	Monohydroxylation	Y	Y
M7	Monohydroxylation	Y	N
M8	Monohydroxylation	Y	Y
M9	Monohydroxylation	Y	N
M10	Dihydroxylation	Y	Y
M11	Dihydroxylation	Y	N
M12	Dihydroxylation	Y	N
M13	Dihydroxylation	Y	Y
M14	Dihydroxylation	Y	Y
M15	Dihydroxylation	Y	Y
M16	Hydration	Y	N
M17	Hydration	Y	N
M18	Desaturation	Y	Y
M19	Desaturation	Y	Y
M20	Methylation	Y	N
M21	Oxidative deamination to alcohol	Y	N
M22	Reduction + Acetylation	Y	Y
M23	Monohydroxylation + Desaturation	Y	N
M24	Dihydroxylation + Desaturation	Y	N
M25	Oxidation + Reduction + Acetylation	Y	Y
M26	Glucuronide	Y	N
M27	Monohydroxylation + Glucuronide	Y	Y
M28	Monohydroxylation + Glucuronide	Y	N

“Y” means discovered; “N” means not discovered.

**Table 2:** Metabolites of Etizolam including the biotransformation, formula, accurate *m/z*, retention time, and diagnostic fragment ions and presence in zebrafish, urine of zebrafish, and human liver microsome (HLM).

RT(min)	[M + H]^+^(*m*/*z*)	Formula	Error (ppm)	MS2 Fragments	Zebrafish Homogenate	Urine and Feces of Zebrafish	HLM	References [7,8]
14.74	359.07248	C_17_H_15_ClN_4_OS	−0.863	341, 315, 178, 154	√	√	√	√
17.384	359.07275	C_17_H_15_ClN_4_OS	−0.111	341, 331, 314,300	√	√	√	√
14.54	359.07227	C_17_H_15_ClN_4_OS	−1.448	341, 314, 224, 154	√	√	√	√
16.626	359.07303	C_17_H_15_ClN_4_OS	0.668	341, 286, 138	√	√	√	/
17.82	359.073	C_17_H_15_ClN_4_OS	0.585	341, 258, 238, 223,138	√	√	√	/
15.795	359.07242	C_17_H_15_ClN_4_OS	−1.030	342, 327, 305, 287	/	√	√	/
18.401	359.0723	C_17_H_15_ClN_4_OS	−1.365	341, 314, 114	√	√	√	√
17.176	359.07278	C_17_H_15_ClN_4_OS	−0.028	341, 330, 275, 138	√	√	√	/
16.226	359.07278	C_17_H_15_ClN_4_OS	−0.028	342, 327, 315	/	√	√	/
15.188	375.06754	C_17_H_15_ClN_4_O_2_S	−0.427	357, 343, 314, 178	/	√	/	√
13.648	375.06598	C_17_H_15_ClN_4_O_2_S	−4.586	357, 244, 185, 152	/	√	/	√
10.846	375.06793	C_17_H_15_ClN_4_O_2_S	0.613	344, 315, 282, 246	/	√	/	/
13.407	375.06732	C_17_H_15_ClN_4_O_2_S	−1.013	357, 330, 316, 258	/	√	/	√
17.777	375.06732	C_17_H_15_ClN_4_O_2_S	−1.013	341, 329, 323, 231	√	√	/	/
17.33	375.06781	C_17_H_15_ClN_4_O_2_S	0.293	357, 332, 314, 138	/	√	/	/
14.98	361.08853	C_17_H_15_ClN_4_OS	0.249	344, 332, 314, 281, 179	/	√	√	√
16.492	361.08884	C_17_H_17_ClN_4_OS	1.108	344, 332, 125, 114	/	√	√	√
19.025	341.06207	C_17_H_13_ClN_4_S	−0.088	305, 272, 237	/	√	√	√
17.381	341.0621	C_17_H_13_ClN_4_S	0.000	300, 291, 273, 258	/	√	√	/
23.479	357.09375	C_18_H_17_ClN_4_S	0.644	328, 309, 259, 223, 165	/	√	√	√
15.02	344.06158	C_17_H_14_ClN_3_OS	−0.901	315, 268, 253,	/	√	/	/
17.409	403.09824	C_19_H_19_ClN_4_O_2_S	−1.885	361, 344, 332,	/	√	/	/
15.324	357.0571	C_17_H_13_ClN_4_OS	−0.112	315, 282, 253, 246	√	√	√	√
14.227	373.05265	C_17_H_13_ClN_4_O_2_S	1.608	355, 344, 316, 287	√	√	/	√
18.586	360.05624	C_17_H_14_ClN_3_O_2_S	−1.555	332, 316, 138	√	√	/	/
13.07	519.11017	C_23_H_23_ClN_4_O_6_S	0.405	343, 314, 289	√	√	√	/
12.6	535.10497	C_23_H_23_ClN_4_O_7_S	0.187	359, 341, 315, 282	/	√	/	/
16.06	535.10497	C_23_H_23_ClN_4_O_7_S	0.187	359, 341, 314, 258	√	√	√	/

“√”—presence, “/”—undetected.

## Data Availability

No new data were created or analyzed in this study. Data sharing is not applicable to this article.
